# Novel Roles of Urokinase- and Tissue-Type Plasminogen Activators in Substance Use Disorders: A Narrative Review of Molecular Mechanisms and Translational Perspectives

**DOI:** 10.3390/ijms27167401

**Published:** 2026-08-19

**Authors:** Amine Bahi, Sinclair Steele

**Affiliations:** 1 Basic Medical Sciences Department, College of Medicine, Ajman University, Ajman P.O. Box 346, United Arab Emirates; a.bahi@ajman.ac.ae; 2 Center of Medical & Bio-Allied Health Sciences Research, Ajman University, Ajman P.O. Box 346, United Arab Emirates; 3 Pathological Sciences Department, College of Medicine, Ajman University, Ajman P.O. Box 346, United Arab Emirates

**Keywords:** Plasminogen activator system, urokinase-type plasminogen activator (uPA), tissue-type plasminogen activator (tPA), substance use disorders, synaptic plasticity, brain-derived neurotrophic factor (BDNF), neuroinflammation

## Abstract

The plasminogen activator system (PAS), comprising urokinase-type plasminogen activator (uPA), tissue-type plasminogen activator (tPA), their receptors, and endogenous inhibitors, has been extensively investigated for its established roles in haemostasis, fibrinolysis, vascular remodelling and extracellular matrix (ECM) homeostasis. Increasing evidence indicates that the functions of the PAS extend well beyond the cardiovascular system and have important regulatory roles in neuronal plasticity, synaptic remodelling, neuroinflammation, and neurotrophic signalling. These processes are increasingly recognized as central contributors to the neurobiological adaptations underlying substance use disorders (SUDs). In this narrative review, we critically evaluate the current evidence regarding the involvement of the PAS in SUDs, with particular emphasis on the molecular and cellular mechanisms through which uPA and tPA influence addiction-related neuroplasticity. The available literature is predominantly derived from preclinical studies, while direct clinical evidence remains limited. Experimental findings support roles for uPA and tPA in modulating reward-related circuitry, behavioural sensitization, relapse-like behaviours, and neurotrophic signalling, including potential interactions with brain-derived neurotrophic factor (BDNF)-related pathways, although the relative contributions of plasmin-dependent and plasmin-independent pathways remain incompletely understood. We also discuss the potential involvement of the PAS in neuroinflammatory responses and synaptic remodelling, together with the challenges associated with translating these findings into clinically relevant biomarkers or therapeutic strategies. Finally, we identify important gaps in current knowledge, including the need for independent replication, mechanistic clarification, and well-designed human studies to establish the clinical relevance of PAS dysregulation in addiction. Collectively, the available evidence supports a modulatory role for the PAS in addiction-related neurobiology and provides a rationale for further translational investigation, while highlighting that PAS-directed therapeutic approaches remain experimental and require substantial preclinical and clinical validation.

## 1. Search Strategy and Scope

This review was conducted as a narrative review with the objective of critically synthesizing current knowledge regarding the involvement of the plasminogen activator system (PAS), particularly urokinase-type plasminogen activator (uPA) and tissue-type plasminogen activator (tPA), in the neurobiology of substance use disorders (SUDs). A comprehensive literature search was performed using the PubMed/MEDLINE, Scopus, and Web of Science databases to identify relevant peer-reviewed publications available up to May 2026. Additional publications were identified through manual screening of the reference lists of relevant articles and reviews. The search strategy combined controlled vocabulary and free-text terms related to the PAS and SUD- and addiction-related neurobiology. Representative search terms included “*urokinase-type plasminogen activator*” OR “*uPA*”, “*tissue-type plasminogen activator*” OR “*tPA*”, “*plasminogen activator system*”, “*uPAR*”, “*plasmin*”, “*plasminogen activator inhibitor-1*” OR “*PAI-1*”, together with “*addiction*”, “*substance use disorder*”, “*drug addiction*”, “*cocaine*”, “*amphetamine*”, “*methamphetamine*”, “*morphine*”, “*opioid*”, “*ethanol*”, “*alcohol*”, “*nicotine*”, “*reward*”, “*conditioned place preference*”, “*behavioural sensitization*”, “*relapse*”, “*dopamine*”, “*brain-derived neurotrophic factor*” OR “*BDNF*”, “*synaptic plasticity*”, “*neuroinflammation*”, and related terms.

Original research articles, systematic reviews and authoritative review articles published in English were considered for inclusion. Particular emphasis was placed on studies investigating the molecular, cellular, behavioural, and translational roles of uPA and tPA in addiction-related neuroplasticity. Both preclinical and clinical studies were included to provide a comprehensive overview of the available evidence, although greater emphasis was necessarily placed on preclinical investigations because of the limited number of human studies currently available. Conference abstracts, non-peer-reviewed publications, studies not directly related to SUDs or addiction-related neurobiology, and articles lacking sufficient methodological detail or relevance to the scope of this review were excluded. As this article is a narrative review rather than a systematic review, no formal quality assessment, risk-of-bias evaluation, or quantitative meta-analysis was performed. Instead, the available literature was critically appraised to identify areas of agreement, conflicting findings, methodological limitations, translational challenges, and important knowledge gaps, with particular emphasis on distinguishing mechanistic evidence derived from experimental models from the currently limited evidence available from human studies.

## 2. The Plasminogen Activator System (PAS): A Brief Overview

Urokinase-type plasminogen activator (uPA) and tissue-type plasminogen activator (tPA), while primarily recognized for their roles in fibrinolysis, exert a significant influence on neuronal plasticity and neurotransmission. These functions have increasingly been implicated in the pathophysiology of several neurological and neuropsychiatric disorders through diverse mechanisms involving synaptic plasticity, neurotrophic signalling and extracellular proteolysis. The involvement of uPA and tPA varies across different disorders, underscoring the complexity of their actions and the critical need for further research to clarify their precise roles in each specific condition. This section provides an overview of the uPA and tPA system, encompassing their enzymatic functions, mechanisms of action and primary physiological roles. This establishes a framework for understanding their involvement in key neurological processes.

### 2.1. Enzymatic Function and Mechanisms

uPA and tPA are closely related serine proteases that constitute the core enzymatic components of the PAS, a tightly regulated proteolytic cascade essential for vascular and extravascular homeostasis [1,2]. Their primary biochemical function is the conversion of plasminogen, an inactive circulating zymogen, into plasmin, a broad-spectrum serine protease responsible for fibrinolysis and extracellular matrix (ECM) degradation [3,4]. Through plasmin generation, uPA and tPA enable fibrin clot dissolution, tissue repair and controlled matrix remodelling [3,5]. Because plasmin is a potent protease, its generation is subject to strict spatial and temporal regulation. A central mechanism of control involves plasminogen activator inhibitor-1 (PAI-1), the principal endogenous inhibitor of both activators, which rapidly forms inactive complexes with uPA and tPA and thereby limits plasmin generation [5,6]. Maintenance of the balance between plasminogen activator activity and PAI-1 levels is essential for haemostasis, as disruption of this equilibrium can lead to either excessive fibrinolysis or pathological fibrin accumulation [2,6]. Within the central nervous system, tPA activity is additionally regulated by neuroserpin, a neuron-specific serine protease inhibitor that restricts excessive extracellular proteolysis and contributes to the maintenance of synaptic homeostasis [7]. This additional layer of regulation highlights the tissue-specific mechanisms governing PAS activity and underscores the complexity of its functions beyond the vascular system.

Despite sharing a common substrate and catalytic mechanism, uPA and tPA differ in their modes of localization and activation, conferring distinct functional profiles. uPA primarily mediates cell surface-associated proteolysis through high-affinity binding to its receptor, urokinase-type plasminogen activator receptor (uPAR), which localizes plasmin generation to the pericellular environment [8,9]. This localization facilitates spatially restricted ECM degradation and pericellular proteolysis, thereby promoting tissue remodelling and cellular invasion. In addition to concentrating extracellular proteolytic activity, uPAR functions as an important signalling receptor despite lacking an intracellular signalling domain. Following uPA binding, uPAR interacts with integrins, low-density lipoprotein receptor-related protein-1 (LRP1), epidermal growth factor receptor (EGFR), and G-protein-coupled receptors to activate intracellular signalling cascades. Specifically, uPAR cooperates with integrins and EGFR to trigger focal adhesion kinase (FAK)/Src proto-oncogene tyrosine kinase (Src)-extracellular signal-regulated kinase 1/2 (ERK1/2) and phosphoinositide 3-kinase (PI3K)-protein kinase B (Akt) signalling [10], while LRP1 functions as a major regulator of uPAR-dependent Rac family small GTPase 1 (Rac1) activation [11]. These plasmin-independent signalling pathways regulate cytoskeletal remodelling, neurite extension, cell migration, and synaptic organization, indicating that uPA influences cellular function through both proteolytic and non-proteolytic mechanisms. For instance, uPA treatment increases the synaptic expression of N-cadherin (NCAD) by a uPAR-mediated, plasmin-independent mechanism that protects synapses from soluble Aβ-induced damage [12]. In addition, uPA activates the Wingless/Integrated (Wnt)-β-catenin pathway by triggering low-density lipoprotein receptor-related protein-6 (LRP6) phosphorylation via a plasmin-independent mechanism, thereby preserving synaptic integrity in the presence of soluble amyloid-β (Aβ) [13]. Furthermore, activation of the uPA–uPAR complex stimulates the small GTPase Ras homolog family member A (RhoA), a critical mediator of cell migration [14]. In contrast, tPA functions predominantly in the fluid phase, particularly within the intravascular compartment, where its activity is markedly enhanced in the presence of fibrin, thereby restricting proteolysis to sites of clot formation [15].

Additional regulatory mechanisms, including differential expression patterns, post-translational modifications, receptor-mediated localization, and feedback regulation by plasmin, further refine the activity of both enzymes [3,4]. This tightly regulated balance enables uPA and tPA to participate in diverse physiological processes while also rendering the PAS highly responsive to pathological stimuli, including those associated with neurological and neuropsychiatric disorders.

### 2.2. Physiological Roles

Beyond their canonical role in fibrinolysis, uPA and tPA contribute to multiple physiological processes that depend on spatially and temporally regulated proteolysis and controlled ECM remodelling [1,2,3]. Through localized plasmin generation, both enzymes support tissue remodelling, wound healing, and regenerative processes by enabling cell migration and matrix reorganization following injury [4,16].

**Figure 1:**
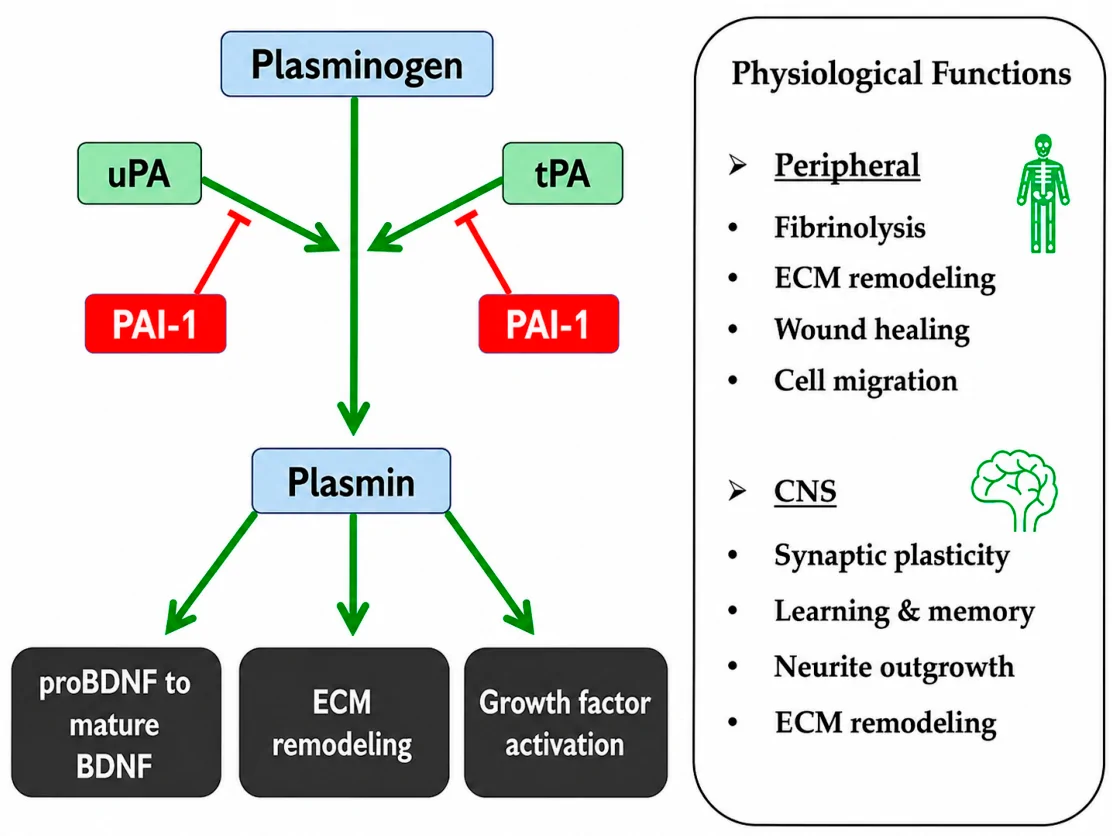
**Overview of the plasminogen activator system and its major physiological functions.** uPA and tPA promote the conversion of plasminogen to plasmin, whereas PAI-1 inhibits uPA and tPA activity. Plasmin contributes to several downstream processes, including the conversion of proBDNF to mature BDNF, ECM remodelling, and growth factor activation. The plasminogen activator system participates in diverse peripheral physiological functions, including fibrinolysis, ECM remodelling, wound healing, and cell migration, as well as central nervous system processes such as synaptic plasticity, learning and memory, neurite outgrowth, and ECM remodelling. **Abbreviations**: CNS: central nervous system; ECM: extracellular matrix; PAI-1: plasminogen activator inhibitor-1; proBDNF: precursor brain-derived neurotrophic factor; tPA: tissue-type plasminogen activator; uPA: urokinase-type plasminogen activator.

In peripheral tissues, plasminogen activators play important roles in physiological processes that require tightly regulated tissue breakdown. In reproductive biology, uPA and tPA are involved in ovulation, follicular rupture, and trophoblast invasion during early pregnancy, processes that depend on precise control of proteolytic activity to permit cellular invasion without excessive tissue destruction [17,18,19]. These examples illustrate how localized plasmin activity enables physiological remodelling while maintaining tissue integrity (Figure 1).

Within the central nervous system (CNS), uPA and tPA have emerged as important modulators of neuronal plasticity and synaptic remodelling. Although they share several physiological functions, accumulating evidence indicates that they often regulate these processes through distinct molecular mechanisms and signalling pathways, a distinction that becomes particularly relevant in the context of addiction-related neuroplasticity. Both enzymes are expressed by neurons and glial cells, and their expression and release are regulated by neuronal activity, including membrane depolarization, glutamatergic neurotransmission, calcium influx, and activity-dependent synaptic stimulation [20,21]. tPA has been particularly well characterized in this context and is implicated in activity-dependent synaptic plasticity through both plasmin-dependent and plasmin-independent mechanisms [7,22,23]. In addition to its proteolytic activity, tPA can modulate neuronal signalling through receptor-mediated mechanisms. In particular, plasmin generated by tPA is capable of activating proteinase-activated receptor-1 (PAR1), thereby influencing neuronal excitability, dopaminergic neurotransmission, and activity-dependent synaptic plasticity [24]. Although the role of PAR1 has been most extensively investigated in experimental models of drug reward, accumulating evidence indicates that this signalling pathway represents an important mechanistic link between PAS activation and neuronal function. Experimental studies have demonstrated that tPA contributes to specific forms of long-term potentiation (LTP) and long-term depression (LTD), cellular processes underlying learning and memory [22,25]. Mechanistically, tPA influences synaptic plasticity through both plasmin-dependent and plasmin-independent pathways. In the plasmin-dependent pathway, tPA converts plasminogen to plasmin, which subsequently degrades ECM components and cleaves proBDNF into mature BDNF, thereby promoting synaptic strengthening and structural remodelling. In contrast, plasmin-independent mechanisms involve direct interactions of tPA with neuronal surface receptors, including N-methyl-D-aspartate (NMDA) receptors and LRP1, enabling modulation of neuronal excitability, calcium signalling, and synaptic transmission independently of extracellular proteolysis. These complementary mechanisms provide the biological basis for many of the addiction-related effects discussed in subsequent sections [21,26]. Although comparatively less studied, uPA also participates in neural remodelling through mechanisms involving cell surface–associated proteolysis and uPAR-mediated signalling [8,9]. Unlike tPA, uPA exerts many of its biological effects through binding to its glycosylphosphatidylinositol (GPI)-anchored receptor, uPAR. Although uPAR lacks an intracellular signalling domain, it forms functional complexes with integrins, low-density lipoprotein receptor-related protein-1 (LRP1), receptor tyrosine kinases and G-protein-coupled receptors, thereby activating intracellular signalling pathways including ERK1/2, PI3K/Akt, focal adhesion kinase (FAK) and Rho-family GTPases [10,27]. These signalling cascades regulate cytoskeletal organization, neurite extension, cell migration and synaptic remodelling independently of plasmin generation, highlighting important non-proteolytic functions of the uPA–uPAR system within the CNS. Experimental studies further support a complementary role for uPA in shaping neural circuits through cell surface-associated proteolysis and receptor-mediated signalling, although its contribution remains less extensively characterized than that of tPA [28,29].

As in peripheral tissues, the activity of uPA and tPA in the CNS is subject to precise spatial and temporal control, thereby preserving structural and functional integrity. Dysregulation of plasminogen activator activity can disrupt synaptic architecture and neural connectivity, implicating this system in neurological and psychiatric disorders [7,20,23,30]. These physiological roles support the concept that uPA and tPA function as important modulators of structural and functional plasticity. Collectively, these physiological roles provide a strong biological rationale for investigating how dysregulation of the PAS contributes to addiction-related neuroplasticity, although the extent to which these mechanisms operate in human SUDs remains to be established.

Although the PAS has recently been reviewed in the context of mood disorders [31], its roles in SUDs and addiction-related neurobiology have not been comprehensively synthesized. Whereas mood disorders are characterized primarily by dysregulation of affective and stress-related circuits, SUDs involve persistent neuroadaptations within reward-related circuitry driven by repeated exposure to drugs of abuse. Consequently, many of the molecular mechanisms discussed in the present review, including drug-specific regulation of uPA and tPA, conditioned reward, behavioural sensitization, relapse-like behaviour, and dopaminergic signalling, have not previously been comprehensively synthesized within a dedicated addiction-focused review. Accordingly, this review integrates the available evidence across psychostimulants, opioids, nicotine, ethanol and methamphetamine (METH) while critically evaluating the mechanistic, translational and therapeutic implications of PAS signalling in SUDs. Throughout this review, “SUDs” is used when referring to the human clinical disorders, whereas “addiction-related” is retained when describing neurobiological mechanisms, behavioural adaptations, and preclinical experimental outcomes relevant to addiction. Rather than considering uPA and tPA as isolated proteases, we examine how their coordinated yet distinct actions shape addiction-related neuroplasticity across different drug-related behavioural processes and evaluate the extent to which these pathways may be therapeutically exploitable.

## 3. uPA and tPA in Addiction: A Neurobiological Perspective

Accumulating evidence, derived predominantly from preclinical studies, indicates that components of the PAS, including uPA and tPA, contribute to neurobiological processes associated with addiction-related neuroplasticity. Although direct evidence in humans remains limited, experimental studies have identified several molecular and cellular mechanisms through which PAS components modulate reward-related circuitry, synaptic plasticity and behavioural responses to drugs of abuse. Addiction is characterized by persistent neuroadaptations within reward-related brain circuits that contribute to compulsive drug seeking and relapse. Given their established roles in activity-dependent synaptic remodelling and neuronal plasticity, uPA and tPA are well positioned to influence addiction-related neural circuitry. However, the extent to which these mechanisms contribute to the pathophysiology of human SUDs remains an important area of ongoing investigation. The following sections examine the available evidence according to major mechanistic themes before considering the drug-specific roles of uPA and tPA in addiction-related behaviours.

### 3.1. Reward Pathways and Behavioural Sensitization

The mesocorticolimbic dopamine system, and particularly the nucleus accumbens (NAcc), plays a central role in reward processing and the reinforcing properties of drugs of abuse [32,33,34,35]. Within this circuitry, repeated drug exposure induces long-lasting structural and functional changes at synapses, a process that underlies behavioural sensitization and compulsive drug-seeking behaviour [36,37,38,39]. Increasing evidence suggests that the PAS contributes to these neuroadaptations not by initiating dopaminergic neurotransmission itself, but by modulating the synaptic and extracellular mechanisms that stabilize drug-induced plasticity within reward circuits.

Both uPA and tPA are expressed in the NAcc and other reward-related regions, and their expression and activity are dynamically regulated by exposure to drugs of abuse [24,40,41,42,43,44,45,46,47,48]. Experimental studies in rodent models have consistently demonstrated that genetic, pharmacological and viral manipulation of PAS components alters drug-related behavioural outcomes, providing strong mechanistic support for a role of the PAS in addiction-related neuroplasticity. However, these behavioural paradigms model discrete aspects of addiction biology and should not be considered direct equivalents of human SUDs. For example, lentiviral-mediated inhibition of uPA in the NAcc impaired cocaine-induced locomotor stimulation and conditioned place preference (CPP), indicating that endogenous uPA positively regulates cocaine reward. Similarly, increased tPA activity enhances the rewarding effects of psychostimulants such as amphetamine (AMPH) and opioids including morphine. Beyond acute reward, experimental evidence indicates that uPA and tPA contribute to the progressive neurobehavioural adaptations that develop following repeated drug exposure, including locomotor sensitization [38,39]. Behavioural paradigms such as CPP, locomotor sensitization and drug-primed reinstatement model discrete components of addiction-related behaviour, including reward learning, behavioural plasticity and relapse-like behaviour. However, they do not fully capture the multifactorial nature of human SUDs, which are influenced by complex genetic, environmental and psychosocial factors. Genetic or pharmacological disruption of tPA signalling attenuates sensitization to psychostimulants, suggesting that PAS-dependent proteolytic activity contributes to the persistence of drug-induced neuroadaptations [43,44,45]. Collectively, these findings support an important modulatory role for uPA and tPA in preclinical addiction-related behavioural paradigms by influencing plasticity within reward circuits rather than acting as primary drivers of neurotransmission. Notably, the available evidence suggests that these proteases regulate distinct components of addiction-related neuroplasticity, with their relative contributions varying according to the drug class, neural circuit, and behavioural process under investigation. Nevertheless, whether these mechanisms contribute meaningfully to the pathophysiology of human SUDs remains to be established through well-designed translational and clinical studies. This apparent context dependence mirrors their physiological roles in other biological systems, where spatially restricted proteolysis enables precise regulation of cellular function [1,7,23].

### 3.2. Neurobiological Mechanisms

The mechanisms by which uPA and tPA contribute to addiction-related neuroadaptations are multifaceted and likely involve convergent effects on synaptic plasticity, neurotransmitter signalling, and neurotrophic pathways. Rather than acting through a single signalling cascade, the PAS influences multiple interconnected molecular processes that collectively regulate activity-dependent neural remodelling within addiction-relevant circuits. One of the best-characterized mechanisms involves the modulation of dopaminergic neurotransmission within the NAcc. Dopamine release and receptor activation are central to reward learning and motivation, and repeated exposure to drugs of abuse produces persistent alterations in dopaminergic signalling that contribute to behavioural sensitization and other addiction-related neuroadaptations [35,49]. Mechanistic studies have demonstrated that psychostimulant-induced activation of dopamine D1 receptors stimulates rapid activity-dependent secretion of pre-existing tPA through a protein kinase A (PKA)-dependent pathway within the NAcc, thereby increasing extracellular tPA activity without directly regulating tissue-type plasminogen activator gene (*PLAT*) transcription [50]. tPA release is further regulated by neuronal depolarization, glutamatergic neurotransmission, and calcium influx, providing a mechanism through which repeated neuronal activation dynamically regulates extracellular proteolytic activity within reward-related circuits. Mechanistically, tPA contributes to synaptic remodelling through both plasmin-dependent and plasmin-independent pathways. Plasmin generated following tPA-mediated activation of plasminogen promotes ECM remodelling and proteolytic conversion of proBDNF to mature BDNF, thereby facilitating dendritic spine maturation, synaptic strengthening, and structural plasticity. In parallel, tPA can directly modulate NMDA receptor signalling and LRP1-dependent pathways independently of plasmin generation, influencing neuronal excitability and activity-dependent synaptic remodelling. Within addiction-relevant circuits, these complementary mechanisms facilitate synaptic remodelling at glutamatergic and dopaminergic synapses, ultimately contributing to dopamine-dependent behavioural adaptations associated with drug reward and sensitization [46,47,51,52,53,54].

An important downstream consequence of PAS activation involves regulation of BDNF signalling. BDNF is a key regulator of neuronal survival, synaptic strength, and structural plasticity, and its dysregulation has been strongly implicated in addiction-related neuroplasticity [55,56,57,58,59,60]. In broader neurobiological contexts, tPA-mediated plasmin generation promotes the proteolytic conversion of proBDNF to mature BDNF, thereby shifting the balance toward signalling pathways that favor synaptic strengthening and long-term plasticity while limiting pathways associated with synaptic weakening [61,62,63,64,65]. Given the established involvement of BDNF in drug-induced neuroplasticity, PAS-dependent regulation of BDNF processing provides a biologically plausible mechanism through which PAS signalling could influence drug-induced synaptic adaptations. However, direct evidence demonstrating that PAS-dependent proBDNF processing mediates addiction-related behavioural outcomes remains limited, and this mechanism should therefore be considered hypothesis-generating.

Although less extensively studied in addiction models, uPA may also influence addiction-related neuroplasticity through plasmin-independent uPAR signalling. As discussed in Section 2.2, following uPA binding, uPAR interacts with integrins, LRP1 and receptor tyrosine kinases, thereby activating intracellular signalling pathways including ERK1/2, PI3K/Akt, focal adhesion kinase (FAK) and Rho-family GTPases. In particular, activation of FAK together with the Rho-family GTPases RhoA and Rac1 coordinates actin cytoskeletal remodelling, thereby regulating dendritic spine morphology [23,66], neurite extension and synaptic stability. These signalling cascades collectively regulate cytoskeletal dynamics and activity-dependent structural plasticity within neuronal circuits providing a plausible mechanistic framework through which uPA may modulate addiction-related neural remodelling. Complementing these uPA-dependent signalling pathways, the plasmin-dependent actions of tPA further promote ECM remodelling, neurotrophic signalling through BDNF maturation, and PAR1 activation, thereby contributing to activity-dependent synaptic plasticity and neuronal excitability within addiction-relevant circuits [8,9,26,67]. Together, these complementary plasmin-dependent and plasmin-independent mechanisms illustrate the diverse molecular pathways through which PAS signalling may influence addiction-related neuroadaptations. It is also becoming increasingly evident that the relative contributions of uPA and tPA differ between drug classes, suggesting that PAS signalling is both drug-specific and brain-region dependent rather than representing a single common mechanism across all substances of abuse. Rather, the available evidence supports a model in which individual PAS components are differentially recruited according to the neurobiological context, resulting in distinct but partially overlapping contributions to addiction-related neuroplasticity. In addition to their downstream effects, uPA and tPA are themselves dynamically regulated by repeated drug exposure. Psychostimulants, opioids, nicotine and ethanol increase PAS activity through activity-dependent neuronal depolarization, glutamatergic neurotransmission and calcium-dependent secretion of pre-existing protease stores, while repeated exposure can also alter the urokinase-type plasminogen activator gene (*PLAU*) and *PLAT* gene expression through intracellular signalling pathways activated during chronic neuroadaptation [68]. These coordinated mechanisms enable the PAS to respond rapidly to acute neuronal activity while potentially contributing to long-term molecular adaptations associated with repeated drug exposure.

Collectively, preclinical evidence supports a model in which uPA and tPA act as modulators of neural remodelling associated with repeated drug exposure within addiction-relevant circuits. By regulating localized proteolysis, synaptic structure, and neurotrophic signalling, the PAS contributes to the persistence of drug-induced behavioural changes. These findings position uPA and tPA as important components of the molecular framework underlying addiction-related neuroadaptations and provide a strong mechanistic rationale for further translational and clinical investigation.

### 3.3. uPA’s Role in Addiction: A Preclinical Perspective

#### 3.3.1. uPA and Psychostimulant Reward: Cocaine- and AMPH-Related Behavioural Adaptations

**Figure 2:**
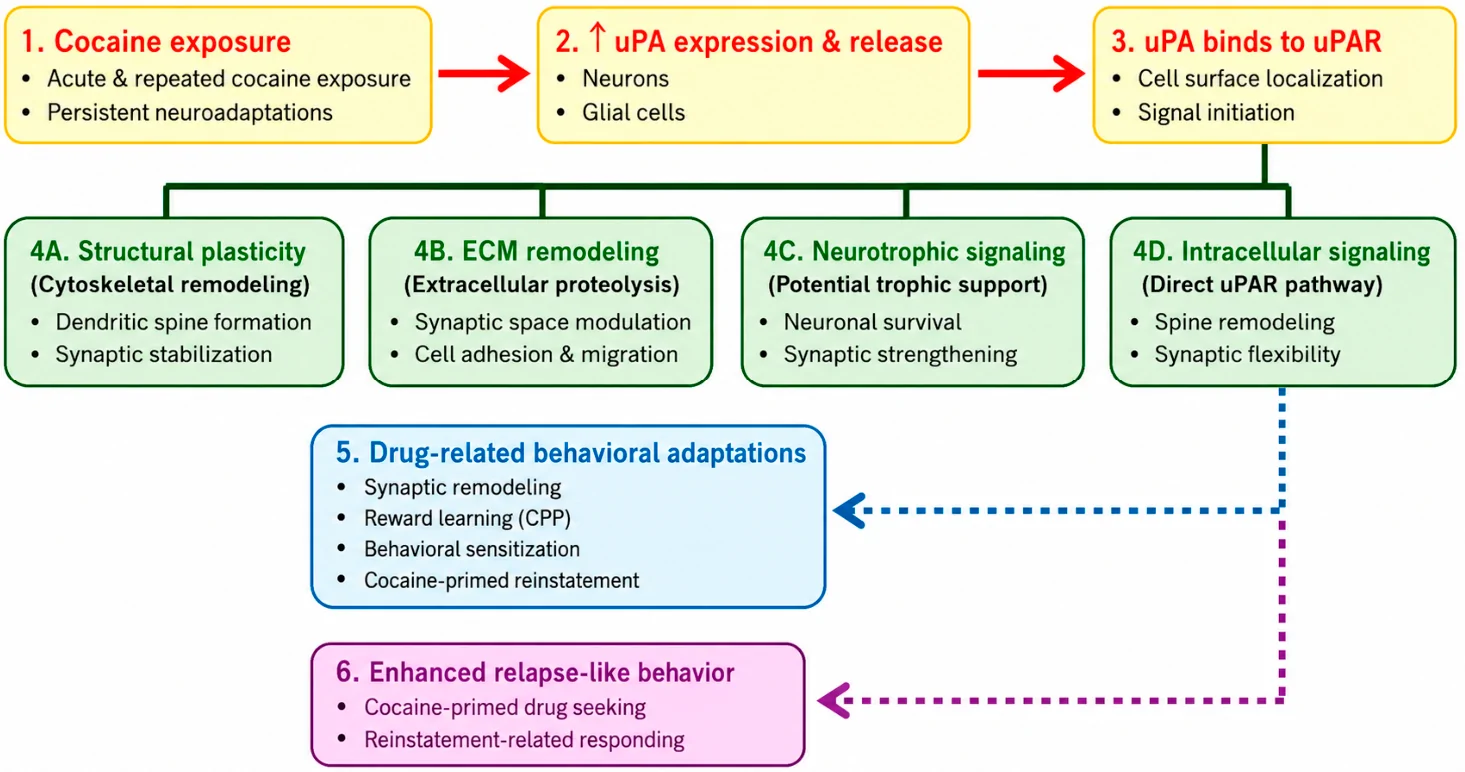
**Proposed mechanisms of uPA-mediated neuroplasticity in cocaine-related behaviours.** Cocaine exposure increases uPA expression and release, promoting uPA binding to its receptor (uPAR) and activation of multiple downstream pathways. These include cytoskeletal remodelling and structural plasticity, ECM remodelling, neurotrophic signalling, and intracellular uPAR-dependent signalling. Together, these mechanisms may contribute to synaptic remodelling and addiction-related neuroadaptations, including conditioned reward, behavioural sensitization, and cocaine-primed reinstatement. Dashed connections indicate proposed mechanisms supported by broader neuroplasticity literature but not yet directly established across all drug classes and behavioural outcomes. **Abbreviations**: CPP, conditioned place preference; ECM, extracellular matrix; uPA, urokinase-type plasminogen activator; uPAR, urokinase-type plasminogen activator receptor.

Preclinical studies employing pharmacological inhibition and genetic manipulation of uPA provide strong evidence that uPA contributes to the rewarding effects of psychostimulants, with the strongest evidence currently available for cocaine. Collectively, these studies indicate that uPA primarily regulates the acquisition of drug-associated neuroplasticity and the development of behavioural adaptations rather than the acute pharmacological actions of psychostimulants. Such effects appear to be mediated through modulation of extracellular proteolysis, synaptic remodelling, and dopaminergic signalling within mesolimbic reward circuits. Although the available evidence for AMPH is comparatively limited, it generally supports a similar, albeit less prominent, role for uPA. Pharmacological inhibition of uPA with B428 significantly reduced cocaine-induced CPP, a widely used behavioural paradigm for assessing drug-associated reward in rodents [69]. These findings support a causal contribution of uPA signalling to cocaine-associated reward in this experimental model. Accumulating preclinical evidence supports an important modulatory role for uPA in cocaine-induced neuroplasticity within the ventral tegmental area (VTA) and the NAcc of the mesolimbic dopaminergic system. In fact, initial studies demonstrated that cocaine robustly upregulates uPA expression in key reward-related regions, including the VTA, NAcc, and hippocampus [70]. Region-specific overexpression of enzymatically active uPA markedly enhanced cocaine-induced locomotor activity [70]. In contrast, expression of a protease-inactive uPA mutant failed to enhance cocaine-induced locomotor activity, indicating that uPA enzymatic activity, rather than expression alone, is required for these behavioural effects [70]. These findings demonstrate that the effect of uPA on cocaine-induced locomotor activity depends on the proteolytic activity of uPA rather than simply increased protein expression. Silencing uPA in the VTA significantly reduced cocaine-induced hyperlocomotion and suppressed expression of the uPA receptor (uPAR), without affecting the endogenous inhibitor PAI-1 [71]. These findings support the concept that the uPA–uPAR axis is an important regulator of dopaminergic responsiveness to cocaine. Beyond locomotion, uPA critically influences conditioned reward and drug-associated learning. Overexpression of uPA in the NAcc enhanced acquisition of cocaine-induced CPP, whereas inhibition of uPA during acquisition abolished CPP formation [69]. Overall, these findings suggest that uPA contributes predominantly to the acquisition of conditioned cocaine-associated memories rather than the expression of established reward memories. These observations indicate that uPA is particularly important during the formation of drug-associated memories, highlighting a role in the early consolidation of addiction-related neuroplasticity rather than the maintenance of established behavioural responses. Comparative studies further demonstrated drug- and protease-specific roles for plasminogen activators. While uPA preferentially enhanced cocaine-induced conditioned responses, tPA selectively potentiated AMPH- and morphine-induced sensitization and CPP [72]. These effects were reversible by doxycycline or small interfering RNA (siRNA)-mediated silencing, underscoring the specificity of extracellular protease signalling in addiction [69,72]. Taken together, the available evidence suggests that cocaine-induced uPA upregulation promotes maladaptive synaptic remodelling within mesolimbic reward circuits, thereby facilitating the neuroplastic changes underlying conditioned reward and other cocaine-related behavioural adaptations. Collectively, the available preclinical studies support an important contribution of uPA to cocaine-induced behavioural adaptations. Current evidence suggests that these effects are mediated by the proteolytic activity of uPA, which likely influences ECM remodelling, synaptic plasticity, and dopamine-dependent neurotransmission within mesolimbic reward circuits, although the precise downstream molecular mechanisms remain incompletely understood. Importantly, the consistency of these findings across complementary pharmacological, viral gene transfer, and genetic manipulation approaches strengthens the evidence for a causal role of uPA in cocaine-induced neuroplasticity, despite the predominance of preclinical models. Despite the consistency of these findings, several limitations should be acknowledged. Much of the available evidence regarding uPA in addiction originates from a relatively limited number of laboratories using closely related rodent models, viral gene transfer approaches and behavioural paradigms. Although these studies provide compelling mechanistic evidence, independent replication across additional laboratories, species and experimental paradigms remains comparatively limited. Furthermore, behavioural measures such as CPP, locomotor sensitization and drug-primed reinstatement model specific components of addiction-related behaviour but do not fully recapitulate the complexity of human SUDs. Consequently, while these experimental models provide valuable mechanistic insights, caution is warranted when extrapolating their findings directly to the clinical manifestations of addiction in humans. Although the available evidence strongly supports a mechanistic role for uPA in cocaine-induced behavioural adaptations, considerably less is known regarding its involvement in responses to other psychostimulants. The role of uPA in AMPH reward, while less extensively studied, also shows a similar pattern. The same comparative study showed that cocaine preferentially induced uPA, whereas AMPH more strongly induced tPA [72]. However, the same study also indicated that uPA overexpression did enhance the rewarding effects of AMPH, albeit to a lesser extent than cocaine. These findings suggest that psychostimulants recruit distinct PAS components despite producing broadly similar behavioural outcomes, supporting the concept that uPA and tPA make complementary, rather than redundant, contributions to drug-induced neuroplasticity. These observations suggest that although uPA contributes to AMPH reward, its role is less prominent than in cocaine-induced behavioural adaptations, indicating drug-specific differences in plasminogen activator signalling within mesolimbic reward circuitry (Figure 2).

#### 3.3.2. uPA and Ethanol: Ethanol-Related Reward and Consumption

Evidence from experimental rodent models suggests that uPA also contributes to ethanol-related reward and reinforcement, although considerably fewer studies have examined alcohol compared with psychostimulant addiction. Employing the same uPA inhibitor, B428, researchers observed a dose-dependent decrease in ethanol intake and preference in mice [73]. This effect was specific to ethanol consumption; the mice did not show altered preference for saccharin or quinine solutions, supporting the absence of a detectable effect on tastant preference under the conditions tested [73]. However, motor function was not directly assessed in this study. Importantly, the CPP paradigm, a well-established measure of drug reward, demonstrated that ethanol-induced CPP was effectively blocked by B428 administration [73]. Collectively, these findings indicate that pharmacological inhibition of uPA attenuates both voluntary ethanol consumption and conditioned reward, supporting a functional role for uPA in alcohol reinforcement, although confirmation in additional experimental paradigms and independent laboratories is still required. Notably, the limited number of available studies precludes definitive conclusions regarding whether the mechanisms underlying ethanol-associated behaviours are conserved across substances of abuse or represent distinct adaptations within PAS signalling. Future comparative studies across different classes of substances of abuse should determine whether ethanol-associated behaviours are mediated by molecular mechanisms shared with psychostimulants or instead involve distinct PAS-dependent signalling pathways underlying drug-specific neuroadaptations. The principal preclinical studies investigating the role of uPA in addiction-related behavioural and neurobiological outcomes are summarized in Table 1.

#### 3.3.3. uPA’s Role in Addiction: The Paucity of Human Clinical Evidence

Although preclinical studies consistently support an important role for uPA in addiction-related neuroplasticity, direct clinical evidence remains limited. To date, few studies have examined whether alterations in uPA expression or activity are associated with SUD severity, craving, relapse vulnerability, or treatment response in individuals with SUDs. Furthermore, it remains unclear whether peripheral measures of uPA accurately reflect its activity within addiction-relevant brain regions such as the VTA, NAcc or prefrontal cortex (PFC). Addressing this and other translational challenges will require well-powered, prospective clinical studies investigating uPA concentrations and activity in cerebrospinal fluid (CSF), blood, and other accessible biological samples, while also determining whether these measures are associated with prospectively assessed clinical outcomes, including SUD severity, duration of abstinence, relapse risk and therapeutic response. Initial cross-sectional case–control studies could establish whether PAS measures differ between individuals with SUDs and appropriately matched controls, but longitudinal designs will be required to determine their potential clinical utility. Such studies should incorporate repeated sampling across active drug use, early and sustained abstinence, relapse, and treatment to establish the temporal stability and within-individual dynamics of PAS measures. Biomarker studies should also evaluate drug-class specificity and control for potential confounding factors that can independently influence peripheral PAS activity, including age, sex, smoking, cardiovascular and metabolic status, inflammatory conditions, and medications. Where feasible, paired CSF and blood measurements would be particularly informative for determining whether peripheral PAS measures reflect central alterations. Finally, candidate biomarkers should be evaluated in independent validation cohorts and tested for incremental predictive value beyond established clinical variables before they can be considered useful for predicting relapse risk or treatment response. Such longitudinal studies will also be essential to determine whether alterations in PAS activity represent stable trait biomarkers, state-dependent indicators of disease progression, or dynamic biological responses to treatment. In addition, species differences in PAS regulation, together with the complexity of human SUDs, require careful consideration when translating findings from rodent models to clinical populations. Consequently, although current preclinical evidence provides a strong mechanistic rationale for investigating uPA in addiction, robust clinical validation is required before uPA can be considered a reliable biomarker or therapeutic target.

### 3.4. tPA’s Role in Addiction: A Multifaceted Involvement

#### 3.4.1. tPA and Opioid Reward: Morphine-Related Behavioural Adaptation

**Figure 3:**
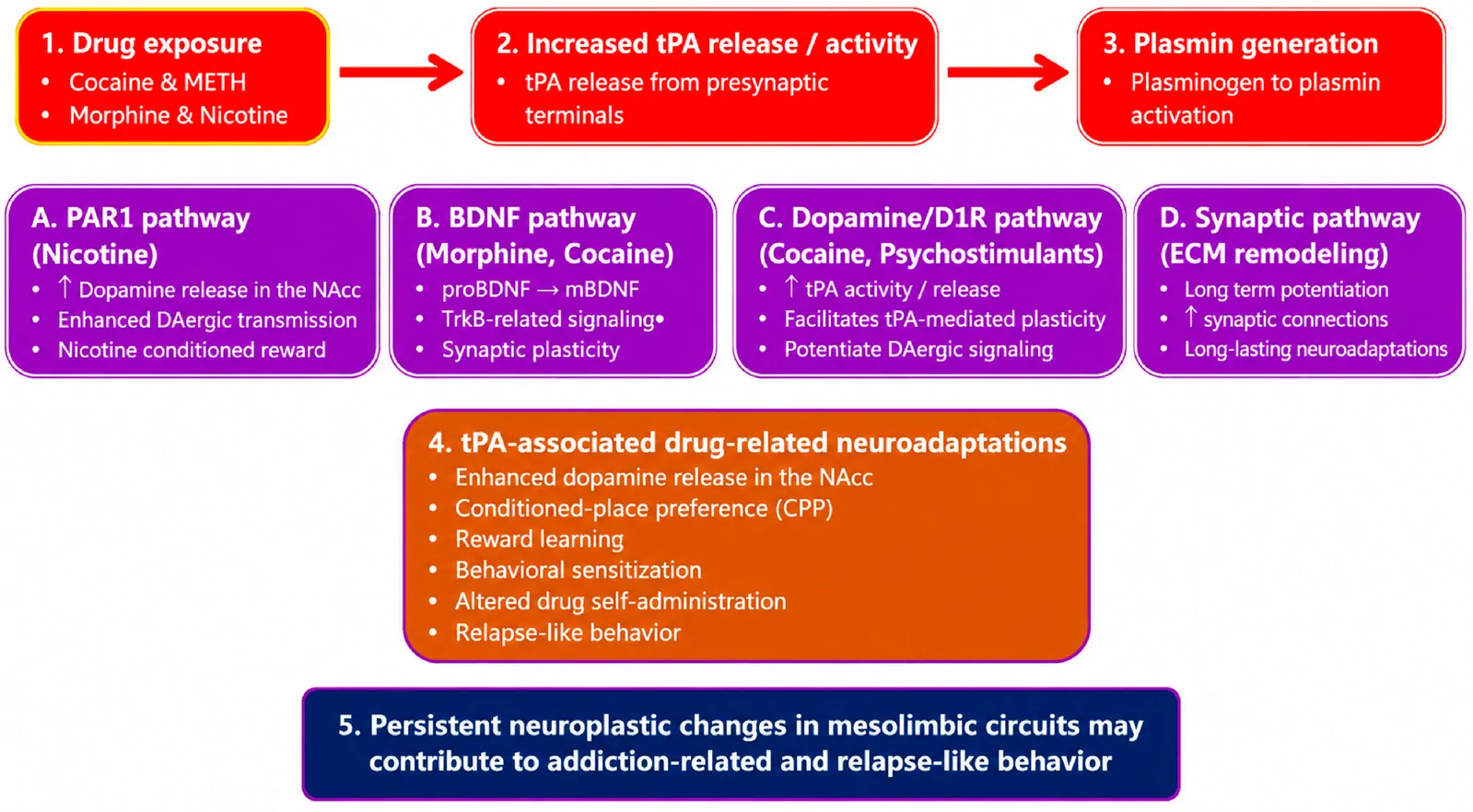
**Proposed mechanisms of tPA-associated drug-related neuroplasticity.** Exposure to drugs of abuse may increase tPA release and activity, promoting plasmin generation and engagement of multiple downstream pathways. These include PAR1-mediated modulation of dopaminergic transmission, D1R-dependent regulation of tPA activity, ECM remodelling associated with synaptic plasticity, and BDNF-related signalling. The BDNF pathway is presented as a proposed neurotrophic mechanism, based on established PAS-dependent BDNF processing in broader neurobiological contexts and evidence implicating BDNF in addiction-related neuroplasticity; however, direct evidence that tPA/plasmin-dependent proBDNF processing mediates drug-related behavioural outcomes remains limited. Collectively, these pathways may contribute to drug-related neuroadaptations, including altered dopamine transmission, conditioned reward, reward learning, behavioural sensitization, altered drug self-administration, and relapse-like behaviour. Persistent neuroplastic changes within mesolimbic circuits may subsequently contribute to the maintenance of these behavioural adaptations. **Abbreviations:** BDNF, brain-derived neurotrophic factor; CPP, conditioned place preference; D1R, dopamine D1 receptor; ECM, extracellular matrix; METH, methamphetamine; NAcc, nucleus accumbens; PAR1, proteinase-activated receptor 1; proBDNF, precursor brain-derived neurotrophic factor; tPA, tissue-type plasminogen activator; TrkB, tropomyosin receptor kinase B.

The role of tPA in opioid-associated reward has been investigated primarily in genetically modified mouse models and viral gene transfer studies. Collectively, the available evidence indicates that tPA positively regulates opioid-induced behavioural plasticity by facilitating both conditioned reward and locomotor sensitization. These experimental approaches have shown that deletion or inhibition of tPA attenuates morphine-induced CPP and hyperlocomotion, whereas tPA overexpression enhances these behavioural responses [72]. Collectively, these findings indicate that tPA facilitates opioid-induced behavioural plasticity, with increased tPA activity enhancing morphine reward and locomotor responses in preclinical models. These observations further support the concept that tPA functions as a modulator of activity-dependent neuroplasticity rather than a direct mediator of opioid neurotransmission. However, these findings are based predominantly on preclinical evidence and require validation in independent experimental systems and human studies (Figure 3).

#### 3.4.2. tPA and Psychostimulant Sensitization: AMPH and Beyond

Similar to uPA, tPA overexpression in the NAcc has been shown to significantly enhance locomotor activity and behavioural sensitization to AMPH and morphine [72,74]. The observation that these effects were fully suppressed by doxycycline, a tetracycline antibiotic that inhibits tPA expression in the experimental model, or by siRNA silencing of tPA provides strong evidence for a causal role [72,74]. Consistent with these findings, METH rapidly increases tPA activity within the NAcc, while genetic deletion of tPA markedly attenuates METH-induced CPP and behavioural sensitization. Importantly, these deficits are rescued by exogenous tPA administration, demonstrating that endogenous tPA is required for the full expression of METH reward and sensitization [43,44]. Studies using tPA-deficient mice further demonstrated enhanced sensitivity to cocaine-induced locomotor activity together with exaggerated responding during non-reinforced periods of cocaine self-administration despite normal acquisition of the operant task [40,75]. Taken together, these studies indicate that tPA regulates multiple behavioural adaptations to psychostimulants, including reward, sensitization, and inhibitory control, through mechanisms likely involving activity-dependent synaptic remodelling and dopaminergic neurotransmission. Collectively, these findings suggest that tPA contributes to multiple psychostimulant-related behavioural adaptations, although its precise contribution appears to vary according to the drug and behavioural outcome examined. Further studies are needed to define the precise molecular pathways underlying these effects and to determine how these mechanisms differ among individual psychostimulants. Additional preclinical evidence supporting the role of tPA across different classes of substances of abuse is summarized in Table 2.

#### 3.4.3. tPA and the Broader Spectrum of Drugs of Abuse: Nicotine and Ethanol

The influence of tPA extends beyond opioids and psychostimulants. Accumulating evidence indicates that tPA is recruited by multiple classes of substances of abuse, although the behavioural and mechanistic consequences of its activation appear to vary according to the drug involved. Direct preclinical evidence demonstrates that METH and nicotine can increase tPA expression, release, or activity within addiction-relevant neural circuits [24,43,44,46,47]. Evidence concerning ethanol is more indirect and derives largely from studies of withdrawal, developmental exposure, or peripheral fibrinolytic responses rather than direct models of ethanol reward or reinforcement. This broad involvement suggests that tPA participates in several conserved neuroplastic mechanisms that are recruited by different classes of substances of abuse, although the relative contribution of these pathways appears to be drug-dependent. Experimental studies demonstrate that the tPA–plasmin system contributes to nicotine-induced reward and dopamine release in the NAcc through activation of proteinase-activated receptor-1 (PAR1) [24,48]. Plasmin generated following tPA activation cleaves and activates PAR1, thereby modulating glutamatergic neurotransmission, neuronal excitability and dopamine release within reward-related circuitry. In addition, tPA has been shown to influence N-methyl-D-aspartate (NMDA) receptor signalling through plasmin-independent mechanisms, providing a complementary pathway through which PAS activation may regulate synaptic plasticity and behavioural responses to substances of abuse [26]. In tPA knockout (tPA−/−) mice, nicotine-induced CPP is significantly attenuated, accompanied by a marked reduction in mesolimbic dopamine release [48,76]. This impairment in dopamine release can be restored by administering exogenous tPA or plasmin directly into the NAcc. Furthermore, acute nicotine exposure increases tPA protein levels and promotes its release into the extracellular space [48,76]. Collectively, these findings indicate that tPA contributes to nicotine-induced reward by coupling extracellular proteolysis to dopamine signalling and synaptic plasticity within mesolimbic reward circuits. Together with findings from psychostimulant and opioid models, these observations support the concept that tPA functions as a regulator of drug-related neuroplasticity across several substance classes, while engaging distinct downstream signalling pathways depending on the substance involved. Additional preclinical evidence supporting the role of tPA across different classes of substances of abuse is summarized in Table 2.

**Table 2:** **Representative preclinical studies investigating the role of tissue-type plasminogen activator (tPA) in addiction-related neuroplasticity.** The table summarizes experimental models, drug-exposure paradigms, tPA-related manipulations, brain regions, control/comparator conditions, principal outcomes, main findings, and study-specific limitations. Sample sizes correspond to the experimental groups relevant to the outcomes summarized and may vary across experiments within individual studies. NR indicates information that could not be verified from the accessible source. **Abbreviations:** AMPH, amphetamine; BDNF, brain-derived neurotrophic factor; CPP, conditioned place preference; D1R, dopamine D1 receptor; D2R, dopamine D2 receptor; DAT, dopamine transporter; ECM, extracellular matrix; FC, frontal cortex; FR2, fixed-ratio 2; GFP, green fluorescent protein; i.p., intraperitoneal; i.v., intravenous; KO, knockout; METH, methamphetamine; NAcc, nucleus accumbens; PAI-1, plasminogen activator inhibitor-1; PAR1, proteinase-activated receptor 1; PKA, protein kinase A; PR, progressive ratio; s.c., subcutaneous; siRNA, small interfering RNA; TH, tyrosine hydroxylase; tPA, tissue-type plasminogen activator; VTA, ventral tegmental area; WT, wild type.

Study	Model	Drug Exposure	tPA Manipulation	Brain Region	Control	Outcomes	Main Findings	Limitations
[40]	tPA−/− and WT mice; *n*/sex by experiment NR	Cocaine; acute/repeated; self-administration, 20 μg/infusion i.v., FR2	Constitutive tPA KO	Global	WT; food reinforcement	Locomotion; sensitization; cocaine self-administration	tPA−/− mice showed greater cocaine sensitivity/sensitization. Self-administration acquisition/rate was unchanged, but KO mice over-responded when cocaine was unavailable; this was absent with food reinforcement.	Global KO; developmental compensation; no region/cell specificity; some *n*/sex details NR.
[44]	Male WT and tPA−/− mice; rats for expression studies; *n* NR	METH; acute/repeated exposure; dose/schedule NR	Constitutive tPA KO; intra-NAcc tPA rescue	NAcc; FC, striatum, hippocampus	WT; vehicle; D1/D2 antagonists	tPA expression/activity; locomotion; sensitization; CPP	Repeated METH increased tPA expression/activity. tPA deletion reduced CPP and sensitization without altering acute hyperlocomotion; intra-NAcc tPA restored sensitization.	*n*/full dosing NR; global KO; CPP/locomotor endpoints; no cell specificity.
[24]	Male ICR, WT, tPA−/− and PAR1−/− mice; CPP *n* = 8–17/group	Nicotine, 0.02 or 0.5 mg/kg s.c.; CPP	tPA/PAR1 deletion; intra-NAcc tPA, plasmin or PAI-1; PAR1 antagonism	NAcc; VTA examined	WT; vehicle; genetic/pharmacological controls	CPP; dopamine release; tPA activity/protein; PAR1 signalling	Nicotine activated NAcc tPA. tPA/plasmin enhanced, whereas PAI-1 or tPA deletion reduced nicotine-evoked dopamine release. PAR1 blockade/deletion attenuated dopamine release; CPP was diminished in tPA−/− and PAR1−/− mice.	Males only; global KO; CPP rather than self-administration; exogenous protease manipulations; cell specificity unresolved.
[45]	Male tPA−/− and WT mice, 8 weeks; total *n* = 120/138	Morphine self-administration; single 24-h session after 8-day operant training	Constitutive tPA KO	Global	WT; saline; food reinforcement	FR2 self-administration; PR breakpoint	tPA−/− mice showed increased FR2 responding but lower PR breakpoints, consistent with reduced morphine reinforcing efficacy. Food and saline responding were unaffected.	Global KO; developmental compensation; no region/cell specificity; atypical single 24-h self-administration session.
[50]	Rodents; *n* NR	Morphine and METH; doses/schedules NR	D1R/D2R agonists/antagonists; PKA inhibition	NAcc	Vehicle; receptor/pharmacological controls	Extracellular tPA activity	D1R activation increased extracellular tPA activity through PKA. Morphine/METH-induced tPA activity was blocked by dopamine receptor antagonism, linking dopaminergic signalling to drug-induced tPA release.	*n*/doses NR; biochemical endpoint; no addiction-related behavioural outcome; pharmacological manipulation.
[50]	Mice; *n* NR	Acute morphine; dose NR	PAR1 antagonism; plasmin rescue in tPA−/− mice	NAcc	WT/tPA−/−; vehicle	Dopamine release; hyperlocomotion; antinociception	PAR1 blockade reduced morphine-induced dopamine release/hyperlocomotion and prevented plasmin rescue in tPA−/− mice, supporting a tPA→plasmin→PAR1 mechanism. Antinociception was unaffected.	*n*/dose NR; acute exposure; no reward/self-administration endpoint; pharmacological PAR1 blockade.
[72]	Male Wistar rats; *n* = 9/group (locomotion); *n* = 6/group (CPP)	Cocaine 20 mg/kg i.p.; AMPH 2 mg/kg i.p.; morphine 10 mg/kg s.c.; acute/repeated	Doxycycline-regulated tPA overexpression; siRNA knockdown	NAcc	GFP; uPA; ± doxycycline	Locomotion; sensitization; CPP; tPA expression/activity	NAcc tPA preferentially enhanced AMPH- and morphine-induced locomotion, sensitization and CPP; effects were reduced by tPA silencing/suppression. Cocaine responses were more strongly associated with uPA.	Males only; small *n*; viral manipulation; CPP/locomotor endpoints; no self-administration.
[74]	Male Wistar rats; *n* = 9/group	AMPH, 5 mg/kg i.p.; CPP conditioning, extinction and reinstatement	Doxycycline-regulated tPA overexpression or siRNA knockdown	NAcc	GFP; ±doxycycline; siRNA	CPP acquisition; extinction; reinstatement; 5-week retention	NAcc tPA promoted AMPH CPP acquisition; overexpression enhanced and knockdown reduced CPP. Acquisition-phase suppression prevented enhanced CPP; overexpression was associated with persistent CPP and robust reinstatement.	Males only; viral manipulation; CPP model; extinction confounded by greater initial CPP; no cell specificity.
[46]	WT, tPA−/− and PAR1−/− mice; *n*/sex NR	METH, 4 mg/kg; repeated high-dose exposure	Constitutive tPA or PAR1 KO	Striatum	WT	TH/DAT; abnormal behaviour; hyperthermia	METH-induced reductions in striatal TH/DAT, abnormal behaviour and hyperthermia were comparable across genotypes, indicating that tPA/PAR1 is not required for METH neurotoxicity.	*n*/sex NR; global KO; neurotoxicity rather than addiction endpoint; no region/cell-specific manipulation.
[47]	WT and tPA−/− mice; *n*/sex NR	METH; acute/repeated exposure; dose/schedule NR	Constitutive tPA KO; intra-NAcc tPA/plasmin	NAcc	WT; vehicle	Dopamine release; laminin degradation	Acute METH-induced dopamine release was preserved in tPA−/− mice, but sensitization of dopamine release was absent. tPA/plasmin enhanced dopamine responses; plasmin degraded laminin, implicating ECM remodelling.	*n*/sex/dosing NR; global KO; exogenous proteases; mechanistic/neurochemical endpoints; no direct reward outcome.
[77]	Male C57BL/6 mice; *n* = 4–16/group depending on experiment	Ethanol, 0.5–2.5 g/kg i.p.; acute, sensitization and CPP	Lentiviral tPA overexpression or shRNA knockdown; recombinant tPA rescue	NAcc; dorsal striatum control	GFP; saline; ±dopamine antagonists	tPA activity; locomotion; sensitization; CPP	Ethanol increased NAcc tPA activity. NAcc tPA overexpression enhanced ethanol locomotion, sensitization and CPP, whereas knockdown attenuated these effects; dorsal-striatal manipulation was ineffective.	Males only; variable/small *n*; viral manipulation; CPP/locomotor endpoints; no voluntary ethanol intake.
[78]	Male C57BL/6 mice; *n* = 14/group	Social defeat; two-bottle choice, 5% and 10% ethanol	Doxycycline-regulated hippocampal tPA overexpression	Hippocampus	CTRL-Sham; DEFEAT-Sham; DEFEAT-tPA; DEFEAT-Doxy	Ethanol intake/preference; depression-like behaviour; BDNF	Social defeat increased ethanol intake/preference and reduced hippocampal tPA/BDNF. tPA overexpression attenuated stress-induced ethanol drinking and depression-like behaviour; doxycycline abolished these effects.	Males only; no non-stressed tPA group; repeated behavioural testing; tPA assessed at mRNA rather than protein/activity level.

#### 3.4.4. tPA’s Mechanism of Action: The proBDNF Pathway

The behavioural effects of tPA described above may involve, at least in part, interactions with neurotrophic signalling. One biologically plausible mechanism through which tPA may contribute to addiction-related neuroplasticity involves the regulation of BDNF signalling [22,79]. BDNF is a key regulator of synaptic plasticity and long-term memory and has been strongly implicated in drug-induced neuroadaptations [59,80]. Dysregulation of BDNF signalling is strongly linked to addiction [80,81,82,83]. In broader neurobiological contexts, tPA converts plasminogen to plasmin, which proteolytically cleaves proBDNF into mature BDNF. This processing is biologically significant because proBDNF and mature BDNF exert distinct, and often opposing, physiological effects. Whereas proBDNF preferentially activates the p75^NTR^ to promote synaptic weakening, dendritic spine retraction and, under certain conditions, apoptotic signalling, mature BDNF preferentially activates tropomyosin receptor kinase B (TrkB), stimulating pathways that support neuronal survival, synaptic strengthening, dendritic spine maturation and long-term potentiation [84]. These established neurobiological actions provide a potential framework through which the tPA–plasmin system could influence the structural and functional neuroplasticity associated with repeated drug exposure.

Importantly, addiction-related evidence provides indirect support for an association between the proBDNF/mature BDNF balance and SUDs. In patients with alcohol dependence, increased plasma proBDNF and decreased mature BDNF were associated with greater recent alcohol consumption and longer drinking history [85]. More directly relevant to the PAS, Liu and colleagues reported that patients with AUD exhibited reduced plasma tPA and mature BDNF together with increased PAI-1 and proBDNF. Following abstinence, tPA and mature BDNF increased, whereas PAI-1 and proBDNF decreased [86], suggesting that alterations in factors regulating extracellular BDNF processing are associated with AUD and may be partially reversible during abstinence. However, these findings are peripheral and associative and do not demonstrate that the observed changes in tPA are responsible for altered proBDNF processing. Moreover, direct studies selectively manipulating tPA/plasmin-dependent proBDNF processing in addiction models remain lacking. Accordingly, although the proBDNF/mature BDNF balance is increasingly supported as a relevant feature of addiction-related neurobiology, its regulation through the tPA–plasmin pathway should currently be regarded as a biologically plausible hypothesis rather than an established mechanism of addiction-related neuroplasticity. Its relative contribution compared with other tPA-dependent signalling pathways, including PAR1 activation and NMDA receptor modulation, therefore remains to be determined.

#### 3.4.5. tPA’s Role in Addiction: The Clinical Landscape

Similar to uPA, direct clinical evidence supporting a role for tPA in human SUDs remains limited despite substantial preclinical evidence implicating this protease in addiction-related neuroplasticity. At present, the available clinical literature is insufficient to determine the clinical significance of altered tPA-related measures in individuals with SUDs or whether such alterations reflect mechanisms relevant to SUD pathophysiology, consequences of chronic drug exposure, or peripheral physiological responses. Human evidence remains sparse and heterogeneous. Most available studies have assessed peripheral fibrinolytic measures or genetic associations; however, recent postmortem transcriptomic analyses provide preliminary evidence of altered CNS regulation of PAS-related genes, including disrupted *PLAT* transcriptional rhythmicity in the DLPFC of individuals who died from opioid overdose. Nevertheless, whether these transcriptional alterations translate into changes in tPA protein abundance or proteolytic activity remains unknown. For example, a genetic association study found no significant association between polymorphisms in the *PLAT* and susceptibility to METH dependence or METH-induced psychosis, suggesting that common genetic variation in *PLAT* is unlikely to represent a major risk factor for addiction [42]. Furthermore, relatively few studies have examined whether alterations in tPA expression or enzymatic activity are associated with SUD severity, craving, relapse susceptibility or treatment response in clinical populations. It also remains uncertain whether peripheral measures of tPA accurately reflect its activity within addiction-relevant brain regions, including the NAcc, VTA and PFC (Table 3).

Future translational studies should investigate whether tPA concentrations or activity in cerebrospinal fluid (CSF), blood or other accessible biological samples are associated with prospectively assessed clinical outcomes, including SUD severity, duration of abstinence, relapse risk and therapeutic response. As discussed above for uPA, rigorous biomarker validation will require longitudinal sampling, careful control of factors influencing peripheral PAS activity, assessment of peripheral-central correspondence where feasible, and replication in independent clinical cohorts. Longitudinal studies incorporating neuroimaging, biochemical biomarkers and behavioural assessments may be particularly valuable for determining whether alterations in PAS activity represent biomarkers of disease course or dynamic responses to treatment.

Successful clinical translation will require overcoming several important challenges, including species differences in PAS regulation, the complexity of human SUDs, and the potential systemic consequences of modulating a protease that plays a central role in haemostasis. Consequently, the clinical utility of targeting tPA as a biomarker or therapeutic intervention remains to be established.

### 3.5. Comparative Analysis of uPA and tPA in Addiction: Distinct Yet Overlapping Roles

While both uPA and tPA contribute to addiction-related neuroplasticity and drug-related behavioural adaptations, the available evidence indicates that they are not functionally interchangeable but instead regulate overlapping yet distinct processes. Their roles appear to differ depending on the specific drug of abuse and the behavioural outcome under consideration. For example, preclinical studies suggest that uPA plays a more prominent role in mediating the rewarding effects of cocaine, whereas tPA is more strongly associated with the rewarding effects of opioids and the development of behavioural sensitization to AMPH and morphine [69,70,71,72,73,74,77,78]. This suggests that the two plasminogen activators may have distinct downstream targets and signalling pathways that contribute to different aspects of the addiction process.

These differences are also reflected in their underlying mechanisms of action. Whereas uPA primarily exerts its effects through uPAR-mediated signalling and localized pericellular proteolysis, tPA acts through both plasmin-dependent and plasmin-independent pathways, including PAR1 activation and modulation of glutamatergic neurotransmission. tPA-mediated plasmin generation can also promote BDNF maturation in broader neurobiological contexts, although the contribution of this pathway to addiction-related behavioural adaptations remains to be directly established. Rather than representing parallel mechanisms, these signalling pathways appear to converge on the common regulation of synaptic plasticity while differentially shaping drug-specific neuroadaptations. Although both proteases ultimately influence synaptic remodelling and behavioural plasticity, the available evidence suggests that they engage partially distinct molecular networks across different drugs and behavioural outcomes.

Despite these differences, both proteases converge on the regulation of synaptic plasticity and ECM remodelling, processes that are fundamental to addiction-related neuroadaptations [88,89,90]. Current evidence suggests that the relative contribution of each protease is determined by the specific drug, neural circuit, and stage of addiction being examined rather than by a universal hierarchy of PAS signalling. Their relative contributions appear to vary according to the drug, brain region, and experimental context, and further studies are required to determine whether their actions are complementary, independent, or synergistic.

An additional distinction lies in the current state of translational evidence. Although both uPA and tPA have emerged as promising mechanistic regulators of addiction-related neuroplasticity, their relative contributions to human SUDs remain undefined owing to the scarcity of clinical and translational studies. Consequently, it remains uncertain whether the experimental findings observed in rodent models accurately reflect the pathophysiology of human SUDs or whether modulation of PAS signalling will ultimately prove therapeutically beneficial in clinical settings.

Importantly, the strength of evidence supporting these protease-specific effects is not uniform across drug classes. For uPA, the most developed preclinical evidence concerns cocaine, for which pharmacological inhibition, region-specific overexpression, protease-inactive mutants, and gene-silencing approaches have been used to examine conditioned reward, locomotor responses, and reinstatement-like behaviour. Evidence for uPA involvement in ethanol-related behaviours is more limited and relies predominantly on pharmacological inhibition, whereas its contribution to AMPH-associated behaviours has been examined in comparatively few studies. For tPA, the strongest evidence is available for morphine, METH, and nicotine, where genetic deletion, viral overexpression, and/or rescue approaches support roles in specific behavioural and neurochemical outcomes. Evidence concerning tPA in cocaine-related behaviours is comparatively limited and suggests effects that may differ from those observed with other psychostimulants. Accordingly, apparent differences between uPA and tPA across drug classes should be interpreted cautiously, because much of the available literature derives from a relatively small number of research groups using related rodent models and experimental paradigms. Independent replication across laboratories, species, brain regions, and complementary behavioural and molecular approaches will therefore be essential to establish the generalizability of these protease- and drug-specific effects.

Taken together, the available evidence suggests that the PAS should not be viewed as a single molecular pathway contributing uniformly to addiction. Rather, uPA and tPA appear to regulate distinct yet partially overlapping aspects of addiction-related neuroplasticity in a drug-, brain region-, and context-dependent manner. Collectively, the available evidence supports the concept that uPA and tPA make complementary, rather than redundant, contributions to addiction-related neuroplasticity, with their relative importance varying according to the drug, neural circuit, and behavioural process under investigation. Importantly, this emerging framework suggests that future therapeutic strategies may need to target specific PAS components according to the neurobiological mechanisms underlying individual SUDs rather than broadly modulating the PAS as a whole. However, these conclusions are based predominantly on preclinical studies employing diverse experimental paradigms, making direct comparisons challenging. Future investigations using standardized behavioural models, multiple species, and independent replication will be essential to determine whether these proteases represent common regulators of addiction-related plasticity or instead exert drug- and circuit-specific functions.

### 3.6. uPA and tPA in the Broader Context of Addiction: Synaptic Plasticity, Neuroinflammation and Neurotrophic Factors

The roles of uPA and tPA in addiction extend beyond their direct effects on reward and sensitization. Accumulating evidence indicates that these proteases participate in a broader network of biological processes that may contribute to addiction-related neuroplasticity. Their involvement in synaptic plasticity is well established in broader neurobiology, whereas their interactions with neuroinflammatory and neurotrophic pathways represent potentially relevant mechanisms whose specific contributions to addiction remain incompletely established. The following sections summarize the current evidence supporting these processes, while distinguishing directly supported addiction-related mechanisms from biologically plausible pathways derived partly from broader neurobiological evidence.

#### 3.6.1. Synaptic Plasticity: The Foundation of Addiction-Related Behaviours

Both uPA and tPA contribute to synaptic plasticity and remodelling through their proteolytic and receptor-mediated signalling activities [62,91,92]. These enzymes are capable of modifying the ECM surrounding synapses, impacting synaptic strength and altering the structure of dendritic spines [93,94,95,96]. These effects are directly relevant to the neuroadaptations that occur during addiction, including the strengthening of drug-associated memories and the development of drug-seeking behaviour [97,98,99,100]. Collectively, these findings support the concept that PAS-dependent regulation of synaptic remodelling may provide a mechanistic framework through which both uPA and tPA influence addiction-related neuroplasticity, despite engaging partially distinct upstream signalling pathways. Further studies are required to define the molecular mechanisms by which uPA and tPA regulate these structural and functional adaptations, and how these processes contribute to addiction-related behaviours.

#### 3.6.2. Neuroinflammation: A Potential Contributor to Addiction

Neuroinflammation is increasingly recognized as a contributor to addiction-related neuroadaptations. However, the direct involvement of uPA and tPA in addiction-associated neuroinflammation remains poorly established. Studies conducted outside the addiction field demonstrate that PAS components can regulate inflammatory and neuroimmune signalling [101,102], providing biological plausibility for a potential interaction between PAS-mediated inflammatory signalling and mechanisms of synaptic plasticity relevant to addiction. Importantly, these findings do not constitute direct evidence that PAS-mediated neuroinflammatory mechanisms operate in SUDs, and this proposed relationship should therefore be considered hypothesis-generating. Future studies are needed to determine whether PAS-dependent inflammatory signalling contributes directly to addiction-related neuroplasticity or represents a secondary consequence of chronic drug exposure.

#### 3.6.3. Neurotrophic Factors: Interplay with BDNF

BDNF is a key regulator of neuronal survival, synaptic plasticity, and dopaminergic function and is strongly implicated in addiction-related neuroplasticity. PAS signalling can regulate BDNF availability and processing in broader neurobiological contexts, providing a potential link between extracellular proteolysis and drug-induced synaptic adaptations. As discussed above, human studies in AUD have identified alterations in peripheral proBDNF/mature BDNF levels and, more recently, concurrent changes in tPA, PAI-1, and BDNF-related signalling during AUD and abstinence. However, these findings are associative and do not establish PAS-dependent regulation of BDNF within addiction-relevant brain circuits. Accordingly, PAS–BDNF interactions should currently be considered a biologically plausible mechanism of addiction-related neuroplasticity rather than an established pathway. Future studies should directly determine whether manipulation of uPA- or tPA-dependent BDNF processing alters synaptic plasticity and drug-related behavioural outcomes in addiction models.

#### 3.6.4. Therapeutic Implications: Targeting uPA and tPA for the Treatment of SUDs

The accumulating preclinical evidence identifying roles for uPA and tPA in addiction-related neuroplasticity provides a rationale for investigating the PAS as a potential therapeutic target. However, no PAS-targeted therapies have yet been evaluated for the treatment of SUDs in clinical trials, and these approaches should currently be regarded as experimental therapeutic strategies requiring further preclinical validation.

Pharmacological inhibition of uPA with B428 has produced effects on drug-related behavioural outcomes in preclinical cocaine and ethanol studies [69,73]. B428 is a competitive small-molecule uPA inhibitor whose biochemical characterization demonstrated substantially greater inhibitory potency toward uPA than toward related proteases, including tPA and plasmin [103,104], supporting its use as an experimental pharmacological tool for investigating uPA-dependent processes. However, important limitations constrain the therapeutic interpretation of these findings. The cited addiction studies did not establish B428 brain penetration or quantify CNS exposure following systemic administration, nor did they directly demonstrate central uPA target engagement. Formal pharmacokinetic and comprehensive safety assessments were also not reported in these addiction studies. Consequently, although B428 provides pharmacological evidence supporting the involvement of uPA in selected drug-related behavioural outcomes, B428 itself should not currently be considered an established therapeutic candidate for SUDs. Further studies establishing CNS exposure, central target engagement, in vivo selectivity, pharmacokinetics, and safety will be required before the therapeutic potential of uPA inhibition can be adequately evaluated.

Similarly, modulation of tPA represents an experimental therapeutic concept based predominantly on preclinical findings involving opioids and AMPH [72,74,77,78]. Because tPA plays an essential physiological role in fibrinolysis, systemic modulation of its activity could disrupt normal haemostasis, potentially increasing the risk of bleeding or thrombosis [2,5,6,105,106,107]. Any therapeutic approach targeting tPA would therefore require strategies capable of selectively modulating relevant CNS signalling while minimizing interference with systemic fibrinolytic function.

An alternative experimental strategy may involve targeting downstream mediators of PAS signalling, including uPAR, PAR1, plasmin, PAI-1, and neuroserpin, rather than directly modulating uPA or tPA. Such approaches could theoretically provide greater mechanistic specificity and potentially reduce the systemic consequences associated with direct modulation of plasminogen activators. For example, selective modulation of PAR1 or uPAR signalling may influence addiction-related neuroplasticity without substantially interfering with physiological fibrinolysis. However, these downstream targets remain largely unexplored in the context of SUDs, and their therapeutic relevance is currently hypothesis-generating. Rigorous pharmacological characterization, demonstration of CNS exposure and target engagement, and assessment of efficacy and safety across independent preclinical models will be required before clinical translation can be considered. Future studies should also define the temporal and cell type-specific regulation of PAS signalling across drug-related behavioural processes, including acquisition, continued drug use, withdrawal, and reinstatement-related behaviour, to determine when and how individual PAS components might be most effectively targeted while minimizing off-target effects.

#### 3.6.5. Future Directions

The growing global burden of SUDs underscores the urgent need for a deeper understanding of the molecular mechanisms underlying addiction and for the development of more effective therapeutic strategies. The accumulating evidence reviewed here identifies the PAS as a potential mechanistic contributor to addiction-related neuroplasticity, but its translational potential remains largely unexplored. Because the available evidence is derived predominantly from preclinical studies employing diverse behavioural paradigms and experimental approaches, it currently provides a strong mechanistic framework and translational rationale rather than definitive support for clinical application.

Several important challenges must now be addressed before these findings can be translated into addiction medicine. The highest priority is to establish whether the mechanistic findings observed in experimental models are relevant to individuals with SUDs. Foremost among these is the need for well-designed clinical studies to determine whether alterations in uPA or tPA activity correlate with SUD severity, relapse vulnerability, treatment response, or long-term clinical outcomes. In parallel, translational studies should establish whether the molecular mechanisms identified in experimental models are conserved in the human brain and determine whether they represent common pathways across SUDs associated with different substance classes or drug-specific adaptations. Integrating molecular analyses with neuroimaging, electrophysiological approaches, and longitudinal behavioural assessments may further clarify how PAS signalling contributes to neuroplastic changes associated with SUD progression, abstinence, and recovery.

Future mechanistic studies should further define how PAS signalling interacts with dopamine neurotransmission, glutamatergic plasticity, ECM remodelling, neuroimmune signalling, and neurotrophic pathways within addiction-relevant brain circuits. Rather than functioning as isolated biological processes, these pathways are likely to interact dynamically, although the nature and functional significance of these interactions remain to be established in addiction models. Particular emphasis should be placed on identifying cell type-specific and brain region-specific mechanisms, determining the temporal regulation of PAS activity across different phases of drug exposure and drug-related behavioural adaptation, and establishing the relative contributions of plasmin-dependent and plasmin-independent signalling pathways.

Finally, any future clinical translation of PAS-targeted approaches will require strategies that achieve high CNS specificity while preserving the essential physiological roles of uPA and tPA in fibrinolysis, vascular homeostasis, and tissue remodelling. Given the distinct yet overlapping functions of uPA and tPA described throughout this review, future therapeutic approaches may also need to selectively target individual PAS components rather than broadly modulating the pathway as a whole. Future investigations should therefore evaluate downstream signalling components, including uPAR, PAR1, plasmin, neuroserpin, and PAI-1, as experimental therapeutic targets that may offer greater mechanistic specificity than direct modulation of uPA or tPA. Addressing these knowledge gaps through integrated preclinical and clinical research will ultimately determine whether modulation of the PAS can be translated into meaningful therapeutic advances for individuals with SUDs.

#### 3.6.6. Strengths, Limitations and Translational Challenges

The current literature provides converging preclinical evidence implicating the PAS in addiction-related neuroplasticity, while also revealing important limitations that constrain interpretation and clinical translation. A major strength of the available literature is the consistent demonstration, across multiple preclinical studies using complementary experimental approaches, that uPA and tPA regulate behavioural responses to several classes of substances of abuse through modulation of synaptic plasticity, ECM remodelling, and dopaminergic neurotransmission within mesolimbic reward circuits. The integration of pharmacological inhibition, genetic knockout models, viral gene transfer approaches, and behavioural paradigms such as conditioned place preference, locomotor sensitization, self-administration, extinction, and reinstatement has provided converging evidence supporting a causal contribution of PAS signalling to selected drug-related behavioural outcomes in preclinical models.

Nevertheless, several important limitations should be acknowledged. First, the available evidence remains overwhelmingly preclinical, with relatively few studies directly examining PAS dysregulation in individuals with SUDs. Consequently, the translational relevance of many mechanistic findings remains uncertain. Second, much of the experimental literature originates from a limited number of research groups using closely related rodent models, behavioural paradigms, and viral manipulation strategies, highlighting the need for independent replication across laboratories, species, and experimental systems. Third, considerable heterogeneity exists among studies with respect to the substances of abuse investigated, brain regions targeted, experimental manipulations employed, and behavioural outcomes assessed, making direct comparisons challenging. Differences in species, sex, drug exposure paradigms, and behavioural endpoints further complicate cross-study comparisons and may contribute to some of the apparent inconsistencies reported across the literature. Furthermore, many behavioural assays capture only selected components of addiction-related behaviour and cannot fully reproduce the complexity of human SUDs. Finally, although accumulating evidence implicates multiple downstream signalling pathways, including uPAR, PAR1, plasmin, neuroserpin, and PAI-1, the hierarchical relationships among these signalling cascades, their interactions with established addiction-related molecular networks, and the precise molecular mechanisms linking PAS activation to long-term synaptic remodelling and drug-related behavioural adaptations remain incompletely understood. These limitations should be considered when interpreting the mechanistic and translational significance of the available evidence.

Overall, the available evidence supports a modulatory role for the PAS in addiction-related neuroplasticity, particularly in preclinical models. However, the predominance of preclinical evidence, limited independent replication, and scarcity of direct human CNS data currently preclude firm conclusions regarding the clinical relevance of PAS dysregulation or its utility as a biomarker or therapeutic target in SUDs.

## Figures and Tables

**Table 1:** **Representative preclinical studies investigating the role of urokinase-type plasminogen activator (uPA) in addiction-related neuroplasticity.** The table summarizes experimental models, drug-exposure paradigms, uPA manipulations, brain regions, control/comparator conditions, principal outcomes, main findings, and study-specific limitations. Sample sizes correspond to the experimental groups relevant to the outcomes summarized and may vary across experiments within individual studies. **Abbreviations:** B428, uPA inhibitor; BEC, blood ethanol concentration; CPP, conditioned place preference; CPu, caudate-putamen; GFP, green fluorescent protein; i.p., intraperitoneal; NAcc, nucleus accumbens; PAI-1, plasminogen activator inhibitor-1; siRNA, small interfering RNA; uPA, urokinase-type plasminogen activator; uPAR, urokinase-type plasminogen activator receptor; VTA, ventral tegmental area; WT, wild type.

Study	Model	Drug Exposure	uPA Manipulation	Brain Region	Control	Outcomes	Main Findings	Limitations
[70]	Male Wistar rats; *n* = 6	Cocaine, 15 mg/kg i.p.; acute, repeated and sensitization; binge: 30 mg/kg × 4	Lentiviral WT-uPA or inactive uPA mutant; doxycycline-regulated	VTA, NAcc, hippocampus/ventral subiculum; CPu for expression	Saline; GFP; inactive uPA; ±doxycycline	uPA expression; locomotor activity	Cocaine increased uPA expression. WT-uPA enhanced cocaine-induced locomotion, whereas inactive uPA attenuated it, supporting dependence on uPA proteolytic activity.	Males only; small *n*; viral overexpression; locomotor endpoint only.
[71]	Male Wistar rats; initial *n* = 9, reduced by sequential sacrifice	Cocaine, 15 mg/kg i.p./day × 15 days	Lentiviral uPA overexpression, inactive uPA, uPA-siRNA; B428 (30 mg/kg)	VTA	GFP; inactive uPA; uPA-siRNA; ±doxycycline	Locomotion/sensitization; uPA, uPAR, PAI-1	uPA overexpression enhanced cocaine hyperlocomotion; uPA suppression/silencing attenuated it. uPAR changed in parallel with uPA, whereas PAI-1 did not.	Males only; declining *n*; locomotor endpoint; B428 markedly suppressed locomotion.
[72]	Male Wistar rats; *n* = 9 (locomotion), *n* = 6 (CPP)	Cocaine, 20 mg/kg i.p.; acute/repeated exposure and CPP	Lentiviral uPA/tPA overexpression and gene silencing; doxycycline-regulated	NAcc	GFP; uPA vs. tPA; ±doxycycline	Locomotion; sensitization; CPP; protease expression/activity	uPA preferentially enhanced cocaine-induced locomotion, sensitization and CPP, whereas tPA showed stronger effects with AMPH/morphine.	Males only; small *n*; viral overexpression; NAcc-focused; CPP/locomotor endpoints.
[69]	Male Wistar rats; *n* = 9	Cocaine, 20 mg/kg i.p. during CPP conditioning; cocaine-primed reinstatement	NAcc uPA overexpression; doxycycline suppression; B428	NAcc; dorsal striatum control	GFP; ±doxycycline; B428/vehicle	CPP acquisition/expression; extinction; reinstatement; retention	uPA enhanced CPP. Blocking uPA during conditioning impaired CPP acquisition, whereas suppression during expression did not. Acquisition-phase uPA also enhanced later retention/reinstatement.	Males only; small *n*; viral overexpression; relatively weak control CPP; no self-administration.
[73]	Male C57BL/6 mice; *n* = 6–16 depending on experiment	Ethanol: two-bottle choice (2.5–20%); CPP, 1.5 g/kg i.p.; BEC, 3 g/kg i.p.	B428, 3–30 mg/kg	Systemic; no region-specific manipulation	Vehicle; saline; saccharin/quinine controls	Ethanol intake/preference; CPP; tastants; BEC	B428 reduced ethanol intake/preference and impaired CPP acquisition without altering total fluid/tastant intake or ethanol pharmacokinetics.	Males only; systemic inhibition; no CNS target-engagement verification; no region/cell specificity.

**Table 3:** **Human studies examining the plasminogen activator system and related pathways in substance use disorders and drug-exposure phenotypes.** Available human evidence is summarized according to the substance or clinical population investigated, PAS component or related measure assessed, biological compartment, principal findings, and major interpretative limitations. Evidence is distinguished as peripheral, genetic, or central nervous system (CNS) to emphasize that alterations in circulating PAS components or fibrinolytic activity do not necessarily reflect PAS signalling within addiction-relevant brain regions. Studies examining proBDNF/mBDNF without direct measurement of PAS components are included as indirect evidence relevant to PAS-dependent BDNF processing. Collectively, the available human evidence remains limited and predominantly peripheral and associative, with only preliminary evidence of PAS-related molecular alterations in the human CNS. **Abbreviations:** AUD, alcohol use disorder; BDNF, brain-derived neurotrophic factor; CNS, central nervous system; DLPFC, dorsolateral prefrontal cortex; mBDNF, mature brain-derived neurotrophic factor; METH, methamphetamine; OOD, opioid overdose death; PAI-1, plasminogen activator inhibitor-1; PAS, plasminogen activator system; *PLAT*, tissue-type plasminogen activator gene; proBDNF, precursor brain-derived neurotrophic factor; p75NTR, p75 neurotrophin receptor; SUD, substance use disorder; tPA, tissue-type plasminogen activator; TrkB, tropomyosin receptor kinase B.

Study	Substance/Clinical Population	PAS Component/Measure	Biological Compartment	Main Finding	Interpretation/Key Limitation
[41]	10 male chronic alcohol users undergoing 21 days of alcohol withdrawal	tPA antigen, PAI-1 antigen/activity, tPA/PAI-1 ratio, overall fibrinolytic capacity	Peripheral blood (plasma)	Following 21 days of abstinence, tPA antigen and PAI-1 decreased, whereas the tPA/PAI-1 ratio and overall fibrinolytic capacity increased; increased fibrinolysis was mainly attributed to reduced PAI-1	Demonstrates altered peripheral fibrinolytic regulation during alcohol withdrawal, but does not assess CNS PAS signalling. Small male-only sample and no control group
[87]	Human participants exposed to transdermal nicotine	Acute endogenous tPA release	Peripheral vascular	Examined the short-term effects of transdermal nicotine on acute endogenous tPA release	Assesses peripheral vascular fibrinolytic function rather than CNS tPA signalling or addiction-related neuroplasticity
[42]	185 Japanese individuals with METH abuse/psychosis and 288 healthy controls	Four polymorphisms in candidate genes of the tPA/plasminogen system	Genetic	No significant differences in the examined polymorphisms were identified between individuals with METH abuse/psychosis and controls	Does not assess PAS expression, protein abundance, enzymatic activity, or CNS signalling; negative candidate-gene findings do not exclude non-genetic PAS involvement
[85]	59 patients with alcohol dependence and 37 age- and sex-matched controls	proBDNF and mBDNF; BDNF Val66Met polymorphism	Peripheral blood (plasma)	Plasma proBDNF was increased and mBDNF decreased in alcohol dependence. proBDNF correlated positively, and mBDNF negatively, with recent alcohol consumption and drinking history	Provides indirect evidence relevant to BDNF processing, but PAS components or proteolytic activity were not measured; peripheral findings do not establish CNS BDNF processing
[86]	15 males with AUD, 15 male alcohol non-dependents, and 15 healthy male controls; AUD participants assessed during 2 and 4 weeks of abstinence	tPA, PAI-1, proBDNF, mBDNF, TrkB, p75NTR	Peripheral blood (plasma)	AUD was associated with ↓ tPA, ↓ mBDNF and ↓ TrkB and ↑ PAI-1, ↑ proBDNF and ↑ p75NTR. During abstinence, these alterations shifted in the opposite direction, with ↑ tPA/mBDNF/TrkB and ↓ PAI-1/proBDNF/p75NTR	Provides direct human evidence linking peripheral PAS components with BDNF-related alterations in AUD, but remains associative and peripheral and does not demonstrate tPA/plasmin-mediated proBDNF cleavage within the CNS. Small, male-only sample and potential medication confounding
[82]	Individuals who died from opioid overdose and controls	*PLAT* transcriptional regulation/rhythmicity	CNS: postmortem DLPFC	*PLAT* was among genes showing loss of transcriptional rhythmicity in opioid overdose death	Provides preliminary human CNS evidence of altered regulation of a PAS-related gene, but does not demonstrate changes in tPA protein or enzymatic activity; chronic opioid exposure cannot be separated from acute overdose-related effects

↓ decrease, ↑ increase.

## Data Availability

No new data were created or analyzed in this study. Data sharing is not applicable to this article.

## Abbreviations

The following abbreviations are used in this manuscript:

**Abbreviation**	**Definition**
Aβ	Amyloid-beta
Akt	Protein kinase B
AMPH	Amphetamine
BBB	Blood–brain barrier
BDNF	Brain-derived neurotrophic factor
CNS	Central nervous system
CPP	Conditioned place preference
CSF	Cerebrospinal fluid
ECM	Extracellular matrix
EGFR	Epidermal growth factor receptor
ERK1/2	Extracellular signal-regulated kinase 1/2
FAK	Focal adhesion kinase
GPI	Glycosylphosphatidylinositol
LRP1	Low-density lipoprotein receptor-related protein 1
LRP6	Low-density lipoprotein receptor-related protein 6
LTD	Long-term depression
LTP	Long-term potentiation
MAPK	Mitogen-activated protein kinase
METH	Methamphetamine
NAcc	Nucleus accumbens
NCAD	N-cadherin
NMDA	N-methyl-D-aspartate
PAI-1	Plasminogen activator inhibitor-1
PAR1	Proteinase-activated receptor 1
PAS	Plasminogen activator system
PFC	Prefrontal cortex
PI3K	Phosphoinositide 3-kinase
PKA	Protein kinase A
*PLAT*	Tissue-type plasminogen activator gene
*PLAU*	Urokinase-type plasminogen activator gene
proBDNF	Precursor brain-derived neurotrophic factor
Rac1	Rac family small GTPase 1
RhoA	Ras homolog family member A
siRNA	Small interfering RNA
Src	Src proto-oncogene tyrosine kinase
tPA	Tissue-type plasminogen activator
TrkB	Tropomyosin receptor kinase B
uPA	Urokinase-type plasminogen activator
uPAR	Urokinase-type plasminogen activator receptor
VTA	Ventral tegmental area
Wnt	Wingless/Integrated
